# The new ‘coN’ staging system combining lymph node metastasis and tumour deposit provides a more accurate prognosis for TNM stage III colon cancer

**DOI:** 10.1002/cam4.5099

**Published:** 2022-08-01

**Authors:** Xitao Wang, Wei Cheng, Xiaolin Dou, Fengbo Tan, Shipeng Yan, Zhongyi Zhou, Yuqiang Li, Biaoxiang Xu, Chongshun Liu, Heming Ge, Mengxiang Tian, Fangchun Liu, Liling Li, Sai Zhang, Qingling Li, Haiping Pei, Qian Pei

**Affiliations:** ^1^ Department of General Surgery, Xiangya Hospital Central South University Changsha People's Republic of China; ^2^ Key Laboratory of Molecular Radiation Oncology Hunan Province Changsha People's Republic of China; ^3^ National Clinical Research Center for Geriatric Disorders (Xiangya Hospital) Central South University Changsha People's Republic of China; ^4^ Department of Hepatobiliary Surgery, Hunan Provincial People's Hospital The First Affiliated Hospital of Hunan Normal University Changsha People's Republic of China; ^5^ Department of Cancer Prevention and Control, Hunan Cancer Hospital and The Affiliated Cancer Hospital of Xiangya School of Medicine Central South University Changsha People's Republic of China; ^6^ Department of Gastroenterology, The Third Xiangya Hospital Central South University Changsha People's Republic of China; ^7^ Department of Pathology, Xiangya Hospital Central South University Changsha People's Republic of China; ^8^ Institute of Medical Sciences, Xiangya Hospital Central South University Changsha People's Republic of China

**Keywords:** colon cancer, lymph node metastasis, multicentre, SEER database, stage III, tumour deposit

## Abstract

**Objective:**

Despite controversy over its origin and definition, the significance of tumour deposit (TD) has been underestimated in the tumour node metastasis (TNM) staging system for colon cancer, especially in stage III patients. We aimed to further confirm the prognostic value of TD in stage III colon cancer and to establish a more accurate ‘coN’ staging system combining TD and lymph node metastasis (LNM).

**Methods:**

Information on stage III colon cancer patients with a definite TD status was retrospectively collected from the Surveillance, Epidemiology and End Results (SEER) database between 2010 and 2017. The effect of TD on prognosis was estimated using Cox regression analysis. Maximally selected rank statistics were used to select the optimal cut‐off value of TD counts. The predictive power of conventional N staging and the new coN staging was evaluated and compared by Akaike's information criterion (AIC), Harrell's concordance index (C‐index) and time‐dependent receiver operating characteristic (ROC) curves. Clinicopathological data of stage III colon cancer patients in the Xiangya database from 2014 to 2018 were collected to validate the coN staging system.

**Results:**

A total of 39,185 patients with stage III colon cancer were included in our study: 38,446 in the SEER cohort and 739 in the Xiangya cohort. The incidence of TD in stage III colon cancer was approximately 30% (26% in SEER and 30% in the Xiangya database). TD was significantly associated with poorer overall survival (OS) (HR = 1.37, 95% CI 1.31–1.44, *p* < 0.001 in SEER). The optimal cut‐off value of TD counts was 4, and the patients were classified into the TD0 (count = 0), TD1 (count = 1–3) and TD2 (count ≥ 4) groups accordingly. The estimated 5‐year OS was significantly different among the three groups (69.4%, 95% CI 68.8%–70.0% in TD0; 60.5%, 95% CI 58.9%–62.2% in TD1 and 42.6%, 95% CI 39.2%–46.4% in TD2, respectively, *p* < 0.001). The coN system integrating LNM and TD was established, and patients with stage III colon cancer were reclassified into five subgroups (coN1a, coN1b, coN2a, coN2b and coN2c). Compared with conventional N staging, the coN staging Cox model had a smaller AIC (197097.581 vs. 197358.006) and a larger C‐index (0.611 vs. 0.601). The AUCs of coN staging at 3, 5 and 7 years were also greater than those of conventional N staging (0.6305, 0.6326, 0.6314 vs. 0.6186, 0.6197, 0.6160). Concomitant with the SEER cohort results, the coN staging Cox model of the Xiangya cohort also had a smaller AIC (2883.856 vs. 2906.741) and a larger C‐index (0.669 vs. 0.633). Greater AUCs at 3, 5 and 7 years for coN staging were also observed in the Xiangya cohort (0.6983, 0.6774, 0.6502 vs. 0.6512, 0.6368, 0.6199).

**Conclusions:**

Not only the presence but also the number of TDs is associated with poor prognosis in stage III colon cancer. A combined N staging system integrating LNM and TD provides more accurate prognostic prediction than the latest AJCC N staging in stage III colon cancer.

## Graphical abstract

The coN staging combining TDs and LNMs results in more powerful prognositic prediction than the conventional N staging in stage III colon cancer.

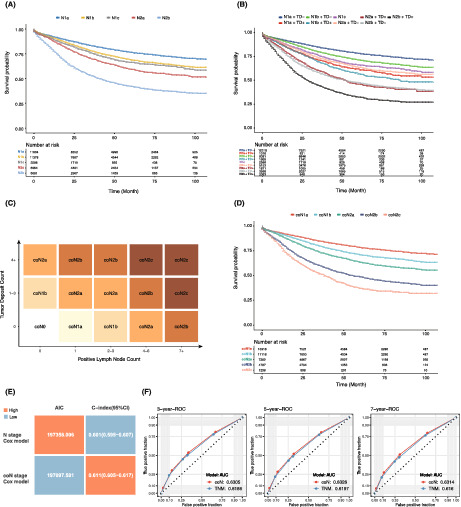

**Highlights**

1. We found that not only the presence but also the number of TDs was associated with poor prognosis in stage III colon cancer, and four proved to be the optimal cut‐off value.
2. We established and validated the ‘coN’ staging system combining TD and LNM and reclassified stage III patients into five subgroups with significantly different survival.
3. The improved predictive power of coN staging will draw much more attention to the latest TD definition in the 8th TNM staging system, which might underestimate the prognostic value of TD to some extent.

## 1 INTRODUCTION

Colorectal cancer (CRC) is the third most common cancer and the second most frequent cause of cancer death worldwide.^1^ Tumour staging systems, which are based on tumour biological behaviour and tested by abundant clinical data, aim to provide an accurate prognostic prediction. The Union for International Cancer Control/American Joint Committee on Cancer (UICC/AJCC) tumour node metastasis (TNM) staging system is a widely accepted cornerstone for CRC treatment decisions and prognosis estimation.^2^

In the TNM system, patients with stage III colon cancer are usually those with regional metastases, as defined by the number of positive lymph nodes and tumour deposit (TD) status. These patients generally receive curative resection followed by systemic adjuvant chemotherapy.^3^ The choice of regimen depends on the patient's regional metastasis state. The IDEA study showed that low‐risk stage III colon cancer patients could choose 3‐month CapeOX adjuvant chemotherapy, which had a similar prognosis but significantly reduced drug toxicity (especially the cumulative neurotoxicity of oxaliplatin) compared to the previously recommended 6‐month CapeOX/FOLFOX.^4^ Individualised chemotherapy on the basis of accurate tumour staging could lead to improved outcomes.

TDs are discrete cancer nodules without lymph structure in the pericolic and mesenteric adipose tissue surrounding CRC.^5^ When lymph node metastases (LNMs) coexist with TD, N1a/b and N2a/b patients are defined exclusively by LNM in the 7th and 8th editions of the TNM staging system. Only in the absence of LNM are stage III patients with TD recorded as ‘N1c’.^6^, ^7^ Considering that TD is present in approximately 20%–30% of stage III patients, ignoring TD in the assessment of patients' regional metastatic state would classify a significant proportion of high‐risk patients into the low‐risk group and lead to inadequate adjuvant treatment8. Several studies have reported underestimation of TD in the prognosis prediction of colon cancer.^8^, ^9^, ^10^ Therefore, more extensive and in‐depth studies are urgently needed to determine the value of TD in the staging of colon cancer.

In this study, we aimed to evaluate the prognostic value of TD in stage III colon cancer by analysing Surveillance, Epidemiology and End Results (SEER) data. A more accurate N staging system was established by incorporating TD and LNM and was validated using multicentre data from China.

## 2 MATERIALS AND METHODS

### 2.1 Patients and data collection from the SEER database

**FIGURE 1:**
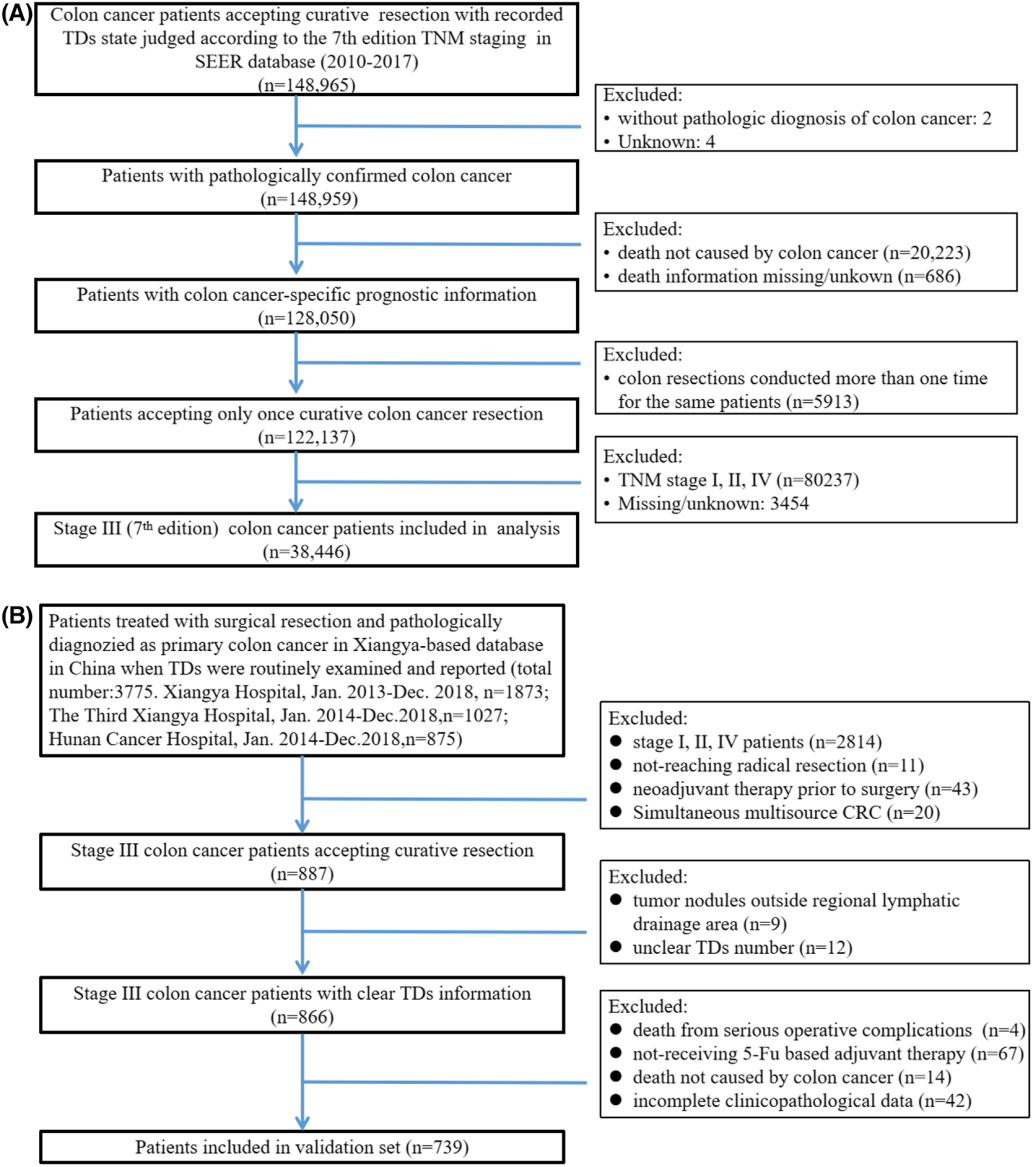
Flowcharts of SEER and Xiangya data. (A) Extraction flowchart of SEER data. (B) Flowchart showing the recruitment process of patients in our multicentre database used as the validation set in our study.

Stage III patients with TD records and survival information between 2010 and 2017 were obtained from the SEER database using SEER*stat software (version 8.3.9.2, https://seer.cancer.gov/). TD was defined according to the 7th edition AJCC TNM staging system. The detailed data extraction procedure is shown in Figure 1A.

Patient characteristics—including age, sex, ethnicity, race, histology, pathological grade, examined lymph nodes, positive lymph nodes, positive lymph node ratio, TNM T stage, TNM N stage, TDs, perineural invasion and location—were included in our analysis. Tumours arising in the caecum, ascending colon, hepatic flexure or transverse colon were classified as right‐sided and those originating in the splenic flexure, descending colon or sigmoid colon were classified as left‐sided.

The endpoint of this study was cancer‐specific overall survival (OS), defined as the time from histological diagnosis to death due to cancer. Time to event was censored if the patient was alive at the last follow‐up.

### 2.2 Patients and data collection in the validation set

We retrospectively collected the clinicopathological data of consecutive pathologically confirmed stage III colon cancer patients undergoing curative resection followed by 5‐FU‐based adjuvant chemotherapy in our CRC database (including CRC data from three different institutions: Xiangya Hospital, Central South University; The Third Xiangya Hospital, Central South University and Hunan Cancer Hospital and The Affiliated Cancer Hospital of Xiangya School of Medicine, Central South University) in China between 1 January 2014 and 31 December 2018, during which the TD status was routinely examined and recorded according to the 7th edition TNM staging system. Although the 8th edition TNM staging system was published on 1 January 2018, because of the controversial definition of TD and the lack of clinical adoption, the pathologists in our study still assessed TDs according to the 7th edition definition in 2018. To further confirm the pathological diagnosis of regional nodes, two pathologists (Q.L. and L.L.) reassessed the sections of nodes and ensured that no regional nodes were merely recorded as a vascular or perineural invasion rather than LNMs or TDs. For patients with TDs but without detailed counts, two pathologists (Q.L. and L.L.) examined the sections and recorded the number of TDs. If any discrepancies arose, agreements were reached by consensus.

The study was approved by the institutional review board (IRB No. 202103496), and the requirement for informed consent was waived due to the retrospective nature of the study. The recruitment and exclusion criteria of the study cohort are presented in Figure 1B.

Data regarding demographics, clinical information, laboratory test values, pathological features, treatment details and outcome information were extracted from medical records and follow‐up databases.

### 2.3 Statistics

Cox proportional hazards regression analysis was performed to assess the association between individual patient characteristics and survival status. Factors with *p* < 0.05 in univariate Cox analysis were included in the final multivariate Cox regression model.^11^ Subgroup analyses were performed for OS‐associated clinical characteristics and are presented as a forest plot. Survival curves were estimated using the Kaplan–Meier method and compared using the log‐rank test with Bonferroni multiplicity adjustment.^12^, ^13^ Differences were considered significant at *p* < 0.05.

The optimal cut‐off point for TD counts was determined using maximally selected rank statistics.^14^ The comparison of staging models covering different parameters was carried out using Akaike's information criterion (AIC) and Harrell's concordance index (C‐index).^15^, ^16^ A smaller AIC value indicated greater goodness‐of‐fit, and a larger C‐index indicated better precision of the predicted outcome. Time‐dependent receiver operating characteristic (ROC) curves and areas under the curve (AUCs) were also calculated to qualify the performance of staging models.^17^

The statistical analysis was performed using R, version 4.1.1 (R Foundation for Statistical Computing, Vienna, Austria), including the ‘tidyverse’, ‘survival’, ‘survminer’ and ‘survival ROC’ packages.^18^ The statistical analysis was directed by an experienced statistician (S.Z.).

## 3 RESULTS

### 3.1 Patient characteristics

A total of 39,185 patients who met the recruitment criteria were included in this study. Among them, 38,446 patients were in the SEER cohort, and 739 patients were in the Xiangya cohort. The baseline characteristics of the patients in both cohorts are listed in Table 1. In the SEER cohort, 11,712 deaths were recorded during a median follow‐up of 4.0 years, and 236 deaths were recorded during a median follow‐up of 4.6 years in the Xiangya cohort. Patients with TNM N1c stage (TD positive but LNM negative) accounted for approximately 7% in the SEER cohort and 10% in the Xiangya cohort. Twenty‐six per cent of SEER cohort patients and 30% of Xiangya cohort patients were TD positive.

**TABLE 1:** Demographic and clinicopathologic characteristics of the recruited patients in the SEER and Xiangya cohorts

Characteristic	SEER, *N* = 38,446	Xiangya, *N* = 739
Age, *n* (%)		
≤65	18,233 (47)	505 (68)
>65	20,213 (53)	234 (32)
Sex, n (%)		
Female	19,879 (52)	300 (41)
Male	18,567 (48)	439 (59)
Ethnicity, *n* (%)		
Non‐Spanish‐Hispanic‐Latino	33,709 (88)	0 (NA)
Spanish‐Hispanic‐Latino	4737 (12)	0 (NA)
Unknown	0	739
Race, *n* (%)		
White	29,505 (77)	0 (NA)
Black	4847 (13)	0 (NA)
Asian or Pacific Islander	3628 (9.5)	0 (NA)
American Indian/Alaska Native	304 (0.8)	0 (NA)
Unknown	162	739
Histology, *n* (%)		
Adenomas and adenocarcinomas	33,892 (88)	699 (95)
Cystic, mucinous and serous neoplasms	4146 (11)	36 (4.9)
Ductal and lobular neoplasms	175 (0.5)	0 (0)
Epithelial neoplasms, NOS	151 (0.4)	2 (0.3)
Other types	82 (0.2)	2 (0.3)
Pathological grade, *n* (%)		
Well/moderately differentiated	27,473 (73)	552 (77)
Poorly differentiated	8346 (22)	165 (23)
Undifferentiated	1993 (5.3)	0 (0)
Unknown	634	22
Examined LN, median (IQR)	18 (14–25)	16 (13–20)
Unknown	106	2
Positive LN, median (IQR)	2.0 (1.0–4.0)	2.0 (1.0–4.0)
Unknown	125	0
LN ratio, median (IQR)	0.12 (0.06–0.25)	0.12 (0.06–0.27)
Unknown	164	2
T stage, *n* (%)		
T1	1634 (4.3)	5 (0.7)
T2	3117 (8.1)	39 (5.3)
T3	24,094 (63)	494 (67)
T4	9466 (25)	201 (27)
Tx	123 (0.3)	0 (0)
Tis	5 (<0.1)	0 (0)
Unknown	7	0
N stage, *n* (%)		
N1a	11,634 (30)	250 (34)
N1b	11,376 (30)	201 (27)
N1c	2586 (7)	75 (10)
N2a	6964 (18)	122 (17)
N2b	5681 (15)	91 (12)
Unknown	205	0
TD, *n* (%)		
Not identified	28,596 (74)	519 (70)
Identified	9850 (26)	220 (30)
Perineural invasion, *n* (%)		
Not identified	29,553 (82)	600 (86)
Identified	6635 (18)	99 (14)
Unknown	2258	40
Location, *n* (%)		
Left	15,028 (40)	429 (59)
Right	22,616 (60)	303 (41)
Unknown	802	7

Abbreviations: IQR, interquartile range; OS, overall survival.

### 3.2 TDs and patient prognosis

**FIGURE 2:**
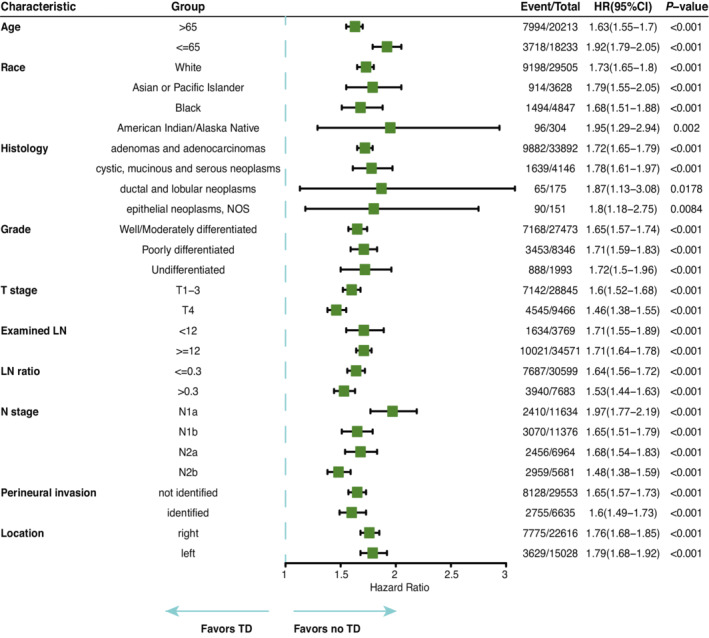
Forest plot for the effect of tumour deposits on overall survival among subgroups.

In the univariate Cox regression analysis of the SEER cohort, the variables significantly associated with OS (*p* < 0.05) were age, ethnicity, race, histology, pathological grade, examined lymph nodes, positive lymph nodes, positive lymph node ratio, T stage, N stage, TDs, perineural invasion and primary tumour location (Table 2). In the multivariate Cox model, including these factors, the presence of TDs was associated with significantly poorer OS (HR = 1.37, 95% CI 1.31–1.44, *p* < 0.001; Table 2). The results of subgroup analysis according to the clinical characteristics significantly associated with OS in the multivariate Cox model were consistent with the overall effect (Figure 2). A similar effect of TDs on OS was also observed in the Xiangya cohort (HR = 1.86, 95% CI 1.36–2.54, *p* < 0.001; Supplementary Table S1).

**TABLE 2:** Univariate and multivariate Cox regression analyses of OS in the SEER cohort

Characteristic	Univariate Cox				Multivariate Cox			
	*N*	Event *N*	HR (95% CI)	*p* value	*N*	Event *N*	HR (95% CI)	*p* value
Age	38,446			**<0.001**	34,596			**<0.001**
≤65		3718	—			3277	—	
>65		7994	2.34 (2.26 to 2.44)			7076	2.20 (2.11 to 2.29)	
Sex	38,446			0.14				
Female		6095	—					
Male		5617	0.97 (0.94 to 1.01)					
Ethnicity	38,446			**<0.001**	34,596			0.38
Non‐Spanish‐Hispanic‐Latino		10,449	—			9271	—	
Spanish‐Hispanic‐Latino		1263	0.88 (0.83 to 0.94)			1082	0.97 (0.91 to 1.04)	
Race	38,284			**<0.001**	34,596			**<0.001**
White		9198	—			8125	—	
Black		1494	0.97 (0.92 to 1.02)			1329	1.18 (1.12 to 1.26)	
Asian or Pacific Islander		914	0.79 (0.74 to 0.84)			813	0.86 (0.80 to 0.93)	
American Indian/Alaska Native		96	0.99 (0.81 to 1.21)			86	1.04 (0.84 to 1.29)	
Histology	38,446			**<0.001**	34,596			**<0.001**
Adenomas and adenocarcinomas		9882	—			8798	—	
Cystic, mucinous and serous neoplasms		1639	1.47 (1.40 to 1.55)			1392	1.11 (1.05 to 1.17)	
Ductal and lobular neoplasms		65	1.53 (1.20 to 1.96)			58	0.95 (0.74 to 1.24)	
Epithelial neoplasms, NOS		90	3.08 (2.50 to 3.78)			74	1.71 (1.35 to 2.15)	
Other types		36	1.97 (1.42 to 2.73)			31	1.41 (0.99 to 2.00)	
Pathological grade	37,812			**<0.001**	34,596			**<0.001**
Well/moderately differentiated		7168	—			6485	—	
Poorly differentiated		3453	1.81 (1.73 to 1.88)			3044	1.26 (1.20 to 1.31)	
Undifferentiated		888	2.14 (1.99 to 2.29)			824	1.38 (1.28 to 1.49)	
Examined LN	38,340	11,655	0.98 (0.98 to 0.98)	**<0.001**	34,596	10,353	0.99 (0.98 to 0.99)	**<0.001**
Positive LN	38,321	11,648	1.07 (1.07 to 1.08)	**<0.001**	34,596	10,353	1.02 (1.01 to 1.03)	**<0.001**
LN ratio	38,282	11,627	8.90 (8.29 to 9.55)	**<0.001**	34,596	10,353	3.26 (2.68 to 3.97)	**<0.001**
T stage	38,439			**<0.001**	34,596			**<0.001**
T1–3		7142	—			6334	—	
T4		4545	2.47 (2.38 to 2.56)			4003	1.95 (1.87 to 2.04)	
Tx		21	0.90 (0.59 to 1.39)			15	1.03 (0.62 to 1.71)	
Tis		1	0.78 (0.11 to 5.56)			1	1.09 (0.15 to 7.74)	
N stage	38,241			**<0.001**	34,596			**<0.001**
N1a		2410	—			2184	—	
N1b		3070	1.35 (1.28 to 1.42)			2702	1.10 (1.04 to 1.17)	
N1c		738	1.49 (1.37 to 1.62)			664	1.16 (1.05 to 1.28)	
N2a		2456	1.87 (1.77 to 1.98)			2195	1.18 (1.11 to 1.27)	
N2b		2959	3.25 (3.08 to 3.43)			2608	1.19 (1.07 to 1.32)	
TD	38,446			**<0.001**	34,596			**<0.001**
Not identified		7759	—			6825	—	
Identified		3953	1.73 (1.66 to 1.80)			3528	1.37 (1.31 to 1.44)	
Perineural invasion	36,188			**<0.001**	34,596			**<0.001**
Not identified		8128	—			7712	—	
Identified		2755	1.73 (1.66 to 1.81)			2641	1.27 (1.22 to 1.33)	
Location	37,644			**<0.001**	34,596			**<0.001**
Left		3629	—			3285	—	
Right		7775	1.55 (1.49 to 1.62)			7068	1.29 (1.24 to 1.35)	

The bold values indicate statistical significance.

Abbreviations: CI, confidence interval; HR, hazard ratio; OS, overall survival.

### 3.3 Cut‐off value for TDs

**FIGURE 3:**
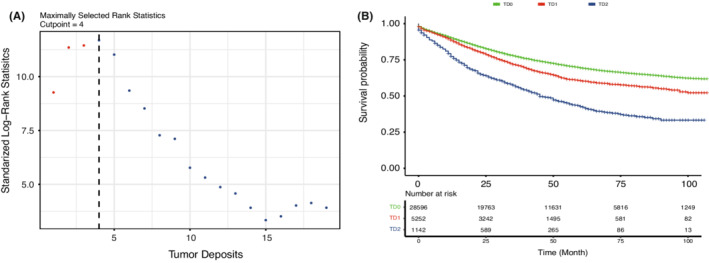
Validation of the cut‐off value of TD number and OS in the TD 0, 1 (count = 1–3) and TD2 (count ≥4) subgroups. (A). The optimal cut‐off value of TD counts was 4 according to maximally selected rank statistics. (B). OS was significantly different among the three subgroups of TDs, with TD0 exhibiting optimal survival and TD2 exhibiting the worst prognosis. TD, tumour deposit; OS, overall survival.

In the SEER cohort, maximally selected rank statistics suggested an optimal cut‐off value of 4 for TD count (Figure 3A). Accordingly, the patients were divided into three groups: TD0 (count = 0), TD1 (count between 1 and 3) and TD2 (count ≥ 4). The survival curves of these three groups were plotted using the Kaplan–Meier method (Figure 3B), and the estimated 5‐year OS rates were 69.4% (95% CI 68.8%–70.0%) for TD0 patients, 60.5% (95% CI 58.9%–62.2%) for TD1 patients and 42.6% (95% CI 39.2%–46.4%) for TD2 patients. The log‐rank test with Bonferroni adjustment showed a significant difference in OS among the three groups (*p* < 0.001; Supplementary Figure S1A). The number of TDs showed a linear effect on OS, with increasing TD numbers associated with decreasing OS.

### 3.4 Construction and validation of the combined N staging

**FIGURE 4:**
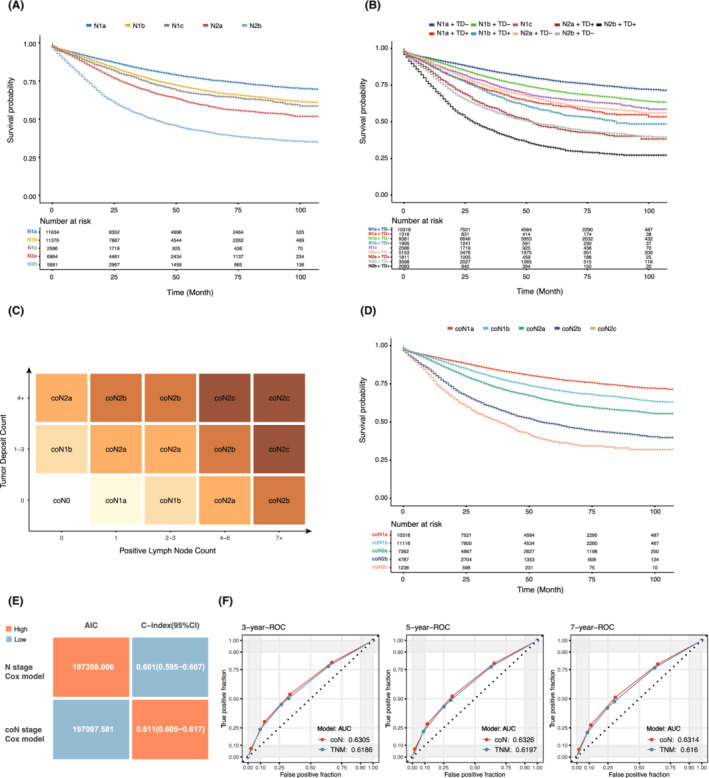
Combining TD and conventional N staging to generate the novel ‘coN’ staging system reflecting tumour regional metastasis in the SEER cohort. (A) Kaplan–Meier survival curves of the five substages according to conventional N staging in the SEER cohort. (B) Kaplan–Meier survival curves when adding TD information to conventional N staging in the SEER cohort, with N1a/b/c and N2a overall survival curves crossing. (C) Combining TD and LNM, the new coN staging strategy represents regional metastasis from two dimensions and classifies stage III patients into five substages. (D) Kaplan–Meier survival curves of the five substages according to coN staging in the SEER cohort. (E) Compared to conventional N staging, the coN staging Cox model had a smaller AIC and a larger C‐index. (F) The AUCs of the ROC curves at 3, 5 and 7 years of coN staging were greater than those of conventional N staging. TD, tumour deposit; LNM, lymph node metastasis; AIC, Akaike's information criterion; C‐index, Harrell's concordance index; AUC, area under the curve; ROC, receiver operating characteristic.

According to the conventional N staging system, patients with different positive lymph nodes and TD statuses can be divided into five subgroups: N1a, N1b, N1c, N2a and N2b. However, the prognostic role of TDs in N1a/b and N2a/b patients has been underestimated. The role of TDs remains ambiguous even in N1c patients, given that the number of TDs affects OS. In the SEER cohort, the estimated 5‐year OS rates of N1b and N1c patients were 69.0% (95% CI 68.0%–70.0%) and 66.2% (95% CI 64.0%–68.4%), respectively (Figure 4A), with no significant difference in OS between the two groups (*p* = 0.127) (Supplementary Figure S1B). When TD status was taken into account, N1a patients with positive TD (N1a + TD+) and N1c patients had a similar prognosis to N2a patients with negative TD (N2a + TD−). The estimated 5‐year OS rates were 62.1% (95% CI 59.0%–65.3%), 66.2% (95% CI 64.1%–68.4%) and 63.7% (95% CI 62.2%–65.2%) for the three groups, without a significant difference in OS among the three groups (*p* > 0.05; Figure 4B and Supplementary Figure S1C). Moreover, N1b patients with positive TD (N1b + TD+) had poorer OS than N2a + TD− patients, with an estimated 5‐year OS of 56.9% (95% CI 54.2%–59.6%, *p* < 0.001; Figure 4B and Supplementary Figure S1C).

Given the prognostic value of TD, we added the TD number to the conventional N staging system to form a novel combined N staging system (coN) (Figure 4C). The coN system has two dimensions: the *x*‐axis for LNM, which is based on conventional N staging, and the *y*‐axis for positive TD number, which is consistent with the previous cut‐off value analysis. Similar to N staging, stage III colon cancer patients were divided into five subgroups by coN staging, and the estimated differences in OS among the five subgroups were significant (*p* < 0.001; Figure 4D and Supplementary Figure S1D).

**FIGURE 5:**
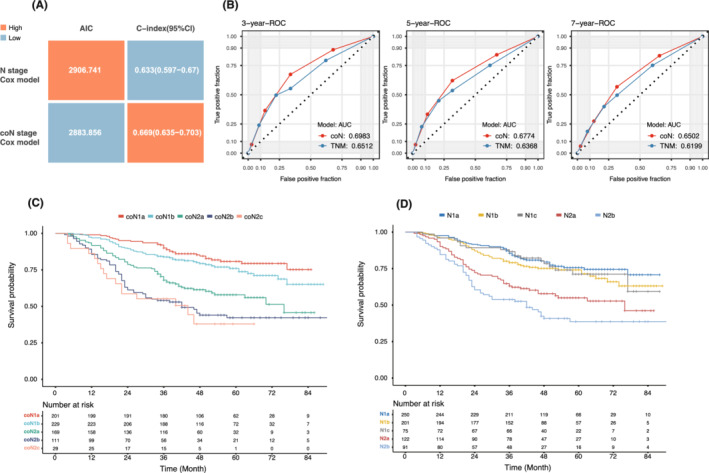
Validation of coN staging in multicentre data from China. (A) Consistent with the SEER cohort, the coN staging Cox model had a smaller AIC and a larger C‐index. (B) The AUCs of the ROC curves at 3, 5 and 7 years of coN staging were also greater than those of conventional N staging in the Xiangya cohort. (C) Kaplan–Meier survival curves of the five substages according to coN staging. (D) Kaplan–Meier survival curves of the five substages according to conventional N staging. AIC, Akaike's information criterion; C‐index, Harrell's concordance index; AUC, area under the curve; ROC, receiver operating characteristic.

Compared with the conventional N staging, the coN staging Cox model had a smaller AIC (197097.581 vs. 197358.006) and a larger C‐index (0.611 vs. 0.601) (Figure 4E). The AUCs of coN staging at 3, 5 and 7 years were also greater than those of conventional N staging (0.6305, 0.6326 and 0.6314 vs. 0.6186, 0.6197 and 0.6160; Figure 4F). Concomitant with the SEER cohort results, the coN staging Cox model of the Xiangya cohort also had a smaller AIC (2883.856 vs. 2906.741) and a larger C‐index (0.669 vs. 0.633; Figure 5A). Larger AUCs at 3, 5 and 7 years for coN staging were also observed in the Xiangya cohort (0.6983, 0.6774 and 0.6502 vs. 0.6512, 0.6368 and 0.6199; Figure 5B). There was less crossover in the estimated survival curves for coN staging than for conventional N staging (Figure 5C,D). These results suggested that the coN staging system had favourable predictive power.

Although the coN staging system was based on OS data due to the lack of disease‐free survival (DFS) information in the SEER cohort, the predictive efficacy of the new staging for DFS was also validated in the Xiangya cohort. Consistent with the above results in the OS analysis, the coN system had a smaller AIC (2658.944 vs. 2666.357), a larger C‐index (0.625 vs. 0.602) and greater AUCs at 3, 5 and 7 years (0.6417, 0.6354, and 0.5987 vs. 0.6147, 0.6065 and 0.5740) in DFS prediction than conventional N staging, indicating the superior predictive power of the coN system (Supplementary Figure S2A,B). However, in general, more cross‐over of estimated survival curves was observed in the DFS prediction compared with that in the OS prediction using either the coN system or conventional N staging model, suggesting that the two prognostic models would be more accurate for OS prediction (Figure 5C,D, Supplementary Figure S2C,D).

## 4 DISCUSSION

In our study, we found that TDs were present in approximately 30% of stage III CC patients, and not only the presence but also the number of TDs (4 as the cut‐off value) was significantly associated with oncological outcomes, generally consistent with the findings of previous investigations.^8^, ^9^, ^10^, ^19^, ^20^, ^21^, ^22^ TDs are defined as tumour foci in the pericolic or perirectal fat in the lymphatic drainage territory, without recognisable residual lymph node tissue.^5^, ^6^ Although the origin of TDs has remained debatable, TDs are widely considered a group of tumour cell entities that have separately or simultaneously grown out of and destroyed different regional histological boundaries such as vascular, lymphatic and neural structures.^23^, ^24^, ^25^ The invaded structures, especially vascular vessels, serve as the main channels for tumour metastasis.^26^, ^27^, ^28^ The breaking of secondary boundaries distant from the primary tumour site also reflects a more profound phenotypic plasticity and more aggressive potential,^23^, ^29^, ^30^ suggesting repeated epithelial‐to‐mesenchymal transition (EMT) and mesenchymal‐to‐epithelial transition (MET), which is required for tumour metastasis.^31^, ^32^ However, in the current TNM staging system, TD is ignored when coexisting with LNM, leading to the loss of important prognosis information. Therefore, it is urgent to re‐evaluate the prognostic value of TD in TNM staging.

The ‘N’ in the TNM staging system, which is based on tumour biology and morphological anatomy, represents regional metastasis. The lymphatic system is believed to be a major metastatic pathway, and mesocolic lymph node invasion is a major kind of regional metastasis in colon cancer.^23^ However, the current N staging fails to accurately reflect regional metastasis when adding TDs to N staging, with reversed or crossed survival curves between N1bTD+ and N2aTD‐ and N2aTD+ and N2bTD−. As mentioned before, TD is another kind of regional metastasis and is more likely to reflect tumour transfer through the vascular system. Since both TD and LNM represent regional metastasis independently, we established a new N staging system combining TD and LNM as quantitative characteristics. Encouragingly, the combined N (coN) staging system generated well‐distinguished substages and had more accurate prognostic predictions than the conventional N staging. According to coN staging, patients with stage III colon cancer were classified into five substages, among which coN1a patients had the best outcome and coN2c patients had the worst prognosis. The predictive power of coN staging was superior to that of conventional N staging, as demonstrated by the AIC, C‐index and AUC values at 3, 5 and 7 years. To validate the stability and universality of coN staging, we tested it in a multicentre database from China and obtained promising reproducible results, suggesting that the coN staging system can be adopted worldwide.

The main goal of accurate tumour staging is to provide a sound foundation for personalised treatment decisions. For low‐risk stage III patients, attenuated regimens seem to be appropriate, with acceptable survival benefits but significantly reduced toxicity. However, for high‐risk patients, such as coN2c patients with only a 37.1% 5‐year OS in the SEER cohort, there is a great need to explore more efficient treatment strategies. More intense adjuvant therapies, such as triple‐drug regimens, chemotherapy combined with immunotherapy or targeted therapy, may lead to improved outcomes.^33^, ^34^ Considering the treatments for locally advanced rectal cancer, neoadjuvant therapy may be another effective choice.^35^ It is noteworthy that the different underlying molecular mechanisms and biological behaviours of LNM and TD may result in quite different responses to traditional chemotherapy regimens. TD‐positive colon cancer patients were able to benefit from FOLFOX adjuvant chemotherapy.^9^, ^22^ However, it remains unclear whether TD‐positive patients can benefit from CapeOX or FOLFIRI.

The prognostic prediction efficacy was elevated when adding TD to the conventional N staging, with the C‐index increasing from 0.601 to 0.611 in the SEER cohort. However, the predictive efficiency remains unsatisfactory, warranting more potential independent prognostic markers other than the N stage, such as molecular signatures (MSI, RAS, BRAF, etc.), pathological features (vascular invasion, tumour budding, etc.), tumour microenvironment characteristics (immune infiltration, extracellular matrix stiffness, etc.) and even imaging characteristics.^36^, ^37^, ^38^, ^39^ It is eagerly expected that stage III patients will receive personalised treatments based on personalised prognostic prediction.

In our study, TD was defined according to the 7th edition TNM staging system and was validated as an independent risk factor for patients with stage III CC. It is worth noting that the definition of TD in the latest 8th edition is different, and it excludes regional nodules with histological evidence of vascular or neural structures.^7^ Since the underlying biological mechanisms of TD remain unclear and TDs are probably the macroscopic, comprehensive and extreme manifestations of vascular or perineural invasions, the new definition raised intense controversy.^5^, ^23^ Due to the insufficient follow‐up time (less than 5 years for the 8th edition definition of TDs implemented from Jan. 2018) and the lack of large‐sample, multicenter, prospective clinical trials, the feasibility and effectiveness of the latest definition of TDs need to be further elucidated in the future. Moreover, a more fundamental biological understanding of TD is the key to improving the definition.

There were several limitations in our study. First, the retrospective data inevitably conferred selection bias, and more abundant information associated with prognosis, such as the status of vascular invasion and perineural invasion, was not widely available. Second, the controversial and transitional definition of TD led to poor uniformity of the TD judgement at different periods and different institutions. Third, larger TDs and increasing numbers of TDs were reported to be significantly associated with more adverse oncological outcomes. However, in our study, the size of the TD was not captured, and some cases were recorded as positive TDs without a specific number and were unavailable for further analysis based on the number of TDs. Fourth, the adjuvant chemotherapy information of patents in the SEER database was missing in our study, and the sample size of our own data was comparatively small. Moreover, the regimens and cycles of adjuvant chemotherapy were vague because of the retrospective nature of the study. Therefore, the influence of chemotherapy regimens and treatment duration on TD‐adjusted substage patients was unclear. Thus, large‐sample, wide‐range, randomised controlled cohort studies with unified judgement criteria for TD are urgently needed to further confirm the significance of TD in stage III colon cancer and to explore personalised treatment strategies for new coN substage patients.

In conclusion, our investigation revealed that not only the presence but also the number of TDs is associated with poor prognosis in stage III colon cancer. A combined N staging integrating LNM and TD provides more accurate prognostic predictions than the latest AJCC N staging in stage III patients.

## AUTHORS' CONTRIBUTIONS

X.W. performed the statistical analyses and participated in manuscript writing. X.D., S.Y., Z.Z., Y.L. and F.L. collected clinicopathological data. F.T., B.X., C.L., H.G. and M.T. conducted follow‐ups and maintained the Xiangya multicentre database. L.L. and Q.L. reviewed the pathology reports and TD status. W.C. and S.Z. rechecked the statistical analyses. Q.L., H.P. and Q.P. designed and directed the entire study. Q.P. and W.C. participated in manuscript writing.

## FUNDING INFORMATION

This study was supported (in part) by the following projects: 1. the Natural Scientific Foundation of China (no. 81702956), 2. the Natural Science Foundation of Hunan Province (nos. 2020JJ4903 and 2020JJ5920), 3. the Construction of Innovative Ability of National Clinical Research Center for Geriatric Disorders (no. 2019SK2335), 4. the Strategy‐Oriented Special Project of Central South University of China (no. ZLXD2017003), 5. the Colorectal Cancer Medical Seed Research Fund of Beijing Bethune Public Welfare Foundation named ‘Effect and mechanism of YAP1 on EGFR resistance in K‐ras wild‐type metastatic colorectal cancer’.

## CONFLICT OF INTEREST

The authors declare that there is no conflict of interest.

## ETHICS STATEMENT

The study was approved by the institutional review board (IRB No. 202103496), and the requirement for informed consent was waived due to the retrospective nature of the study.

## Supporting information


Table S1

Figure S1

Figure S2

## Data Availability

The SEER cohort data that support the findings of this study are openly available in SEER at https://seer.cancer.gov/. The Xiangya cohort data that support the findings of this study are available on request from the corresponding author. The data are not publicly available due to privacy or ethical restrictions.
