# Supplementary material for: The new ‘coN’ staging system combining lymph node metastasis and tumour deposit provides a more accurate prognosis for TNM stage III colon cancer
Source: Cancer Med. 2022 Aug 1;12(3):2538–50. doi: 10.1002/cam4.5099 (PMC9939212; doi:10.1002/cam4.5099)
Supplement: Supplementary file 1 — Table S1 [file CAM4-12-2538-s002.docx]

| **Table S1. Univariate and multivariate Cox regression analyses of OS in the Xiangya cohort** | | | | | | | | | |
| --- | --- | --- | --- | --- | --- | --- | --- | --- | --- |
| **Characteristic** | **Univariate Cox** | | | |  | **Multivariate Cox** | | | |
|  | **N** | **Event N** | **HR (95% CI)^1^** | ***P*-value** |  | **N** | **Event N** | **HR (95% CI)^1^** | ***P*-value** |
| **Age** | 739 |  |  | 0.17 |  |  |  |  |  |
| <=65 |  | 152 | — |  |  |  |  |  |  |
| >65 |  | 84 | 1.21 (0.93 to 1.58) |  |  |  |  |  |  |
| **Sex** | 739 |  |  | 0.75 |  |  |  |  |  |
| Female |  | 96 | — |  |  |  |  |  |  |
| Male |  | 140 | 0.96 (0.74 to 1.24) |  |  |  |  |  |  |
| **Histology** | 739 |  |  | 0.71 |  |  |  |  |  |
| adenomas and adenocarcinomas |  | 220 | — |  |  |  |  |  |  |
| cystic, mucinous and serous neoplasms |  | 14 | 1.35 (0.78 to 2.31) |  |  |  |  |  |  |
| epithelial neoplasms, NOS |  | 1 | 1.47 (0.21 to 10.5) |  |  |  |  |  |  |
| other types |  | 1 | 1.66 (0.23 to 11.8) |  |  |  |  |  |  |
| **Pathological grade** | 717 |  |  | **<0.001** |  | 673 |  |  | 0.23 |
| Poorly differentiated |  | 71 | — |  |  |  | 65 | — |  |
| Well/Moderately differentiated |  | 154 | 0.55 (0.42 to 0.73) |  |  |  | 139 | 0.82 (0.60 to 1.13) |  |
| **Examined LN** | 737 | 234 | 1.01 (0.99 to 1.02) | 0.44 |  |  |  |  |  |
| **Positive LN** | 739 | 236 | 1.14 (1.10 to 1.17) | **<0.001** |  | 673 | 204 | 1.06 (0.96 to 1.17) | 0.29 |
| **LN ratio** | 737 | 234 | 7.48 (4.61 to 12.1) | **<0.001** |  | 673 | 204 | 3.17 (0.72 to 14.0) | 0.13 |
| **T stage** | 739 |  |  | **<0.001** |  | 673 |  |  | **0.002** |
| T1-3 |  | 146 | — |  |  |  | 124 | — |  |
| T4 |  | 90 | 1.85 (1.42 to 2.41) |  |  |  | 80 | 1.59 (1.20 to 2.12) |  |
| **N stage** | 739 |  |  | **<0.001** |  | 673 |  |  | 0.25 |
| N1a |  | 55 | — |  |  |  | 48 | — |  |
| N1b |  | 55 | 1.29 (0.89 to 1.88) |  |  |  | 46 | 1.01 (0.66 to 1.54) |  |
| N1c |  | 19 | 1.13 (0.67 to 1.91) |  |  |  | 16 | 0.77 (0.42 to 1.44) |  |
| N2a |  | 54 | 2.40 (1.65 to 3.50) |  |  |  | 49 | 1.44 (0.85 to 2.43) |  |
| N2b |  | 53 | 3.49 (2.39 to 5.10) |  |  |  | 45 | 1.04 (0.39 to 2.75) |  |
| **TD** | 739 |  |  | **0.007** |  | 673 |  |  | **<0.001** |
| not identified |  | 153 | — |  |  |  | 127 | — |  |
| identified |  | 83 | 1.45 (1.11 to 1.90) |  |  |  | 77 | 1.86 (1.36 to 2.54) |  |
| **Perineural invasion** | 699 |  |  | **0.003** |  | 673 |  |  | **0.035** |
| not identified |  | 176 | — |  |  |  | 164 | — |  |
| identified |  | 42 | 1.71 (1.22 to 2.40) |  |  |  | 40 | 1.48 (1.04 to 2.11) |  |
| **Location** | 732 |  |  | **0.001** |  | 673 |  |  | **<0.001** |
| left |  | 117 | — |  |  |  | 103 | — |  |
| right |  | 114 | 1.53 (1.18 to 1.98) |  |  |  | 101 | 1.80 (1.33 to 2.43) |  |
| ^1^HR = Hazard Ratio, CI = Confidence Interval | | | | |  |  | | | |
