# Supplementary figures and images for: The new ‘coN’ staging system combining lymph node metastasis and tumour deposit provides a more accurate prognosis for TNM stage III colon cancer
Source: Cancer Med. 2022 Aug 1;12(3):2538–50. doi: 10.1002/cam4.5099 (PMC9939212; doi:10.1002/cam4.5099)

A

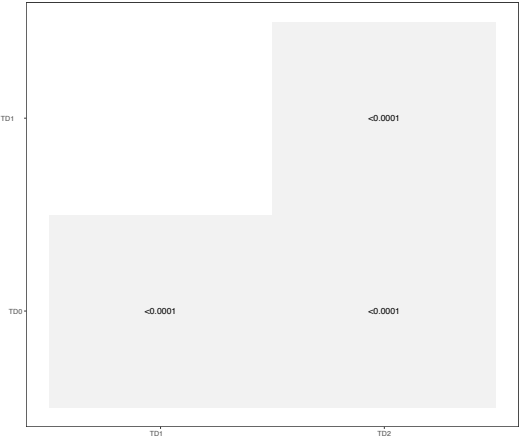

B

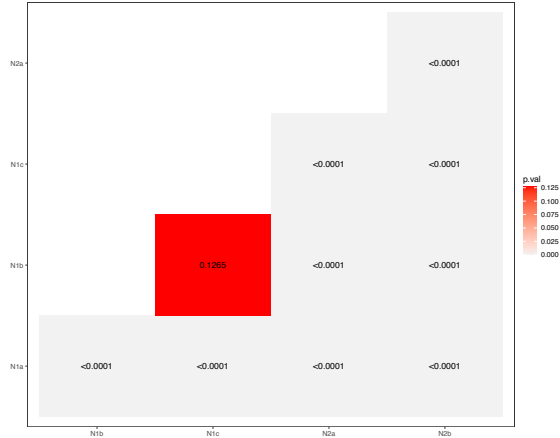

C

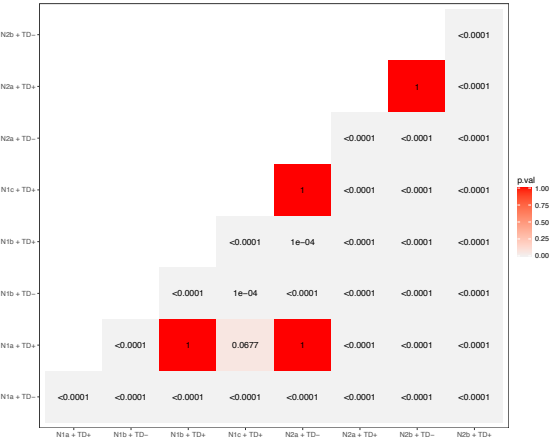

D

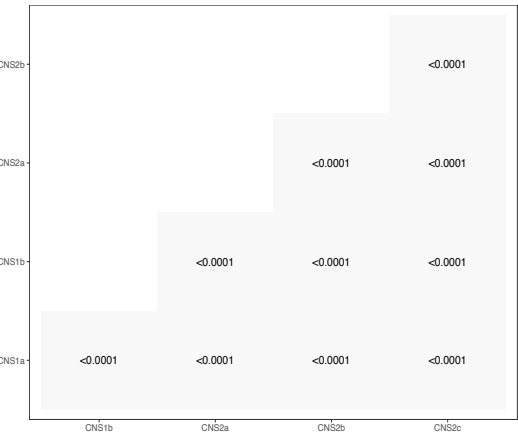

Supplement: Supplementary file 2 — Figure S1 [file CAM4-12-2538-s003.pdf]

A

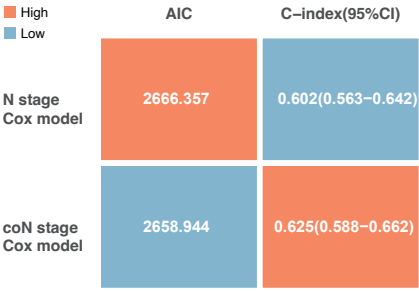

B

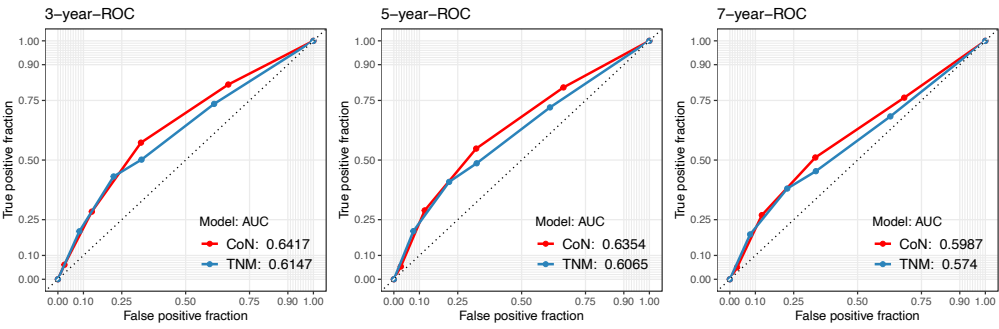

C

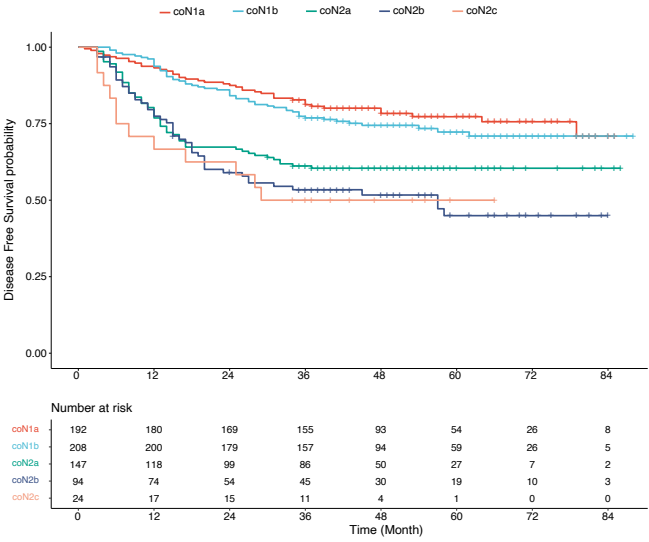

D

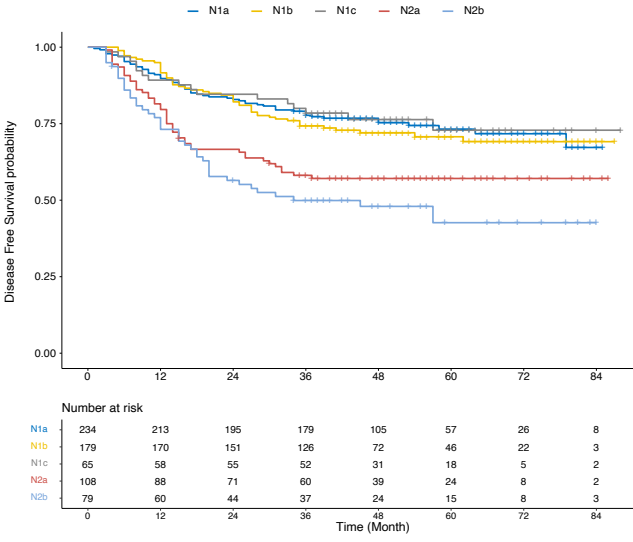

Supplement: Supplementary file 3 — Figure S2 [file CAM4-12-2538-s001.pdf]
